# The design and testing of mini-barcode markers in marine lobsters

**DOI:** 10.1371/journal.pone.0210492

**Published:** 2019-01-24

**Authors:** Ashrenee Govender, Johan Groeneveld, Sohana Singh, Sandi Willows-Munro

**Affiliations:** 1 School of Life Sciences, University of KwaZulu-Natal, Pietermaritzburg, South Africa; 2 Oceanographic Research Institute, Durban, KwaZulu-Natal, South Africa; Tierarztliche Hochschule Hannover, GERMANY

## Abstract

Full-length mitochondrial cytochrome c oxidase I (COI) sequence information from lobster phyllosoma larvae can be difficult to obtain when DNA is degraded or fragmented. Primers that amplify smaller fragments are also more useful in metabarcoding studies. In this study, we developed and tested a method to design a taxon-specific mini-barcode primer set for marine lobsters. The shortest, most informative portion of the COI gene region was identified *in silico*, and a DNA barcode gap analysis was performed to assess its reliability as species diagnostic marker. Primers were designed, and cross-species amplification success was tested on DNA extracted from a taxonomic range of spiny-, clawed-, slipper- and blind lobsters. The mini-barcode primers successfully amplified both adult and phyllosoma COI fragments, and were able to successfully delimit all species analyzed. Previously published universal primer sets were also tested and sometimes failed to amplify COI from phyllosoma samples. The newly designed taxon-specific mini-barcode primers will increase the success rate of species identification in bulk environmental samples and add to the growing DNA metabarcoding toolkit.

## Introduction

Holthuis (1991) provided a detailed systematic catalogue of nearly all the marine lobsters known up to the early 1990s, based solely on the morphology of adult specimens. The traditional classification system used in the catalogue recognized the superfamilies; Nephropoidea (clawed lobsters), Palinuroidea (spiny and slipper lobsters), Eryonoidea (blind lobsters) and the living fossil Glypheoidea within the decapod suborder Macrura Reptantia [1]. More recently, Chan (2010) updated the list of valid species by adding several newly described taxa, and organizing all living marine lobsters into four infraorders: Astacidea, Glypheidea, Achelata and Polychelida. The new (2010) checklist recognized six families, 55 genera and 248 species (with four subspecies) of marine lobsters [2].

Marine lobsters have cryptic early life-history stages. Larvae (called phyllosomas) hatch from eggs carried ventrally on the abdomen of the female and are then dispersed as meroplankton by water movements [3]. Phyllosomas are dorso-ventrally flattened, leaf-like and transparent, and moult through a series of developmental stages of increasing size and morphological complexity [4]. The final phyllosoma stage undergoes a metamorphic molt into a post-larva (or puerulus), which settles on the substrate to begin a benthic existence. Early benthic juveniles are morphologically similar to adult lobsters, and can readily be identified to species level [4]. In contrast, phyllosomas are difficult to distinguish, because they are morphologically cryptic, and have not yet been fully described for all extant taxa [5, 6].

DNA barcoding is now well established as a technique for species identification and discovery [7]. It relies on short, standardized nucleotide sequences (DNA barcodes) as internal species tags and searchable online sequence repositories, such as the Barcode of Life Data Systems (BOLD, www.barcodeoflife.org) and International Nucleotide Sequence Database Collaboration (www.insdc.org). DNA barcoding augments traditional taxonomy methods [8] and is particularly useful for distinguishing cryptic or polymorphic species, such as marine lobsters, and for associating life history stages of unknown identities, such as eggs or larvae, with identifiable adult stages [9].

The DNA barcode used in most animal groups is a 658-base pair (bp) protein-coding region of the mitochondrial cytochrome c oxidase 1 (COI) gene [7]. This region has been successfully used to identify adult lobsters and phyllosomas [10, 11], but amplification success of phyllosomas is often low [12], possibly because of rapid post-capture DNA degradation and fragmentation [13, 14]. Fragmented DNA shorter than the spanning length of the COI primer would hinder amplification [15]. Several studies have consequently relied on 16S rRNA or 18S rRNA gene regions to obtain higher amplification success rates [16–19]. A higher COI amplification success rate can potentially be obtained by designing a shorter informative section of the gene region, as a mini-barcode.

Mini-barcodes need to be sufficiently informative and preferentially target hypervariable DNA regions to accurately delimit species [20, 21]. The reliability of mini-barcodes relies on the presence of a ‘barcode gap’, or the difference between inter- and intraspecific genetic distances within a group of organisms [22, 23]. Several studies have successfully designed and tested mini-barcodes in species from a wide taxonomic range, for example, moths [24], Australian mammals [25] and Indian snakes [26]. Meusnier *et al*. (2008) developed a ‘universal’ mini-barcode from the standard COI gene region to successfully identify a range of mammals, fishes, birds, and insects from archival samples. Nevertheless, a limitation of universal primers is that the most informative portion of the COI region is not the same for all taxa [27]. Hence, taxon-specific primers tend to have higher PCR amplification and sequencing success rates and offer higher discriminating power than universal primers [15].

Advances in next-generation sequencing and metabarcoding encourages the development of primers for taxon-specific mini-barcodes, which will, in turn, improve the efficiency and accuracy of taxon discovery and identification [21], especially in bulk samples such as mixed zooplankton collected from tow-nets. The taxonomic coverage of the primer sets can then be used in a tree-of-life approach in ecosystem biomonitoring [24, 28]. In this study we used an *in silico* method to identify the shortest, most informative portion of the COI gene region in marine lobsters, and used it to design a taxon-specific mini-barcode. The reliability of the mini-barcode as identification tool was tested using DNA barcode gap analysis. The cross-species amplification of the mini-barcode primers was tested on the tissue of a broad range of lobster taxa, including species of spiny- (Palinuridae), clawed- (Nephropidae), slipper- (Scyllaridae) and blind lobsters (Polychelidae). The amplification success of the mini-barcode primers on phyllosomas was compared with that of primers already available in the literature.

## Materials and methods

A total of 350 lobster COI sequences were downloaded from GenBank and BOLD (www.ncbi.nlm.nih.gov/genbank, date accessed: 02-05-2017http://www.boldsystems.org/, date accessed: 02-05-2017) (S1 Spreadsheet). Where available, individuals from different geographical regions were included in the dataset to accommodate potential phylogeographic structure within recognized species. The final dataset included 175 species belonging to 42 genera and 4 families, covering some 71% of known marine lobster species, 76% of the genera and 67% of the families listed by Chan (2010). The sequences were aligned using Clustal X2.1 [29], and optimized manually to ensure homology, using Bioedit 7.2.5 [30]. The numbers of variable- (V) and parsimony informative characters (Pi), and the average nucleotide composition were estimated for the data (full-length and mini-barcode alignments) using MEGA 6.0 [31].

Mini-barcode fragments were estimated using sliding window analysis (SWAN) [32] in the Species Identity and Evolution (SPIDER) [33] package in R (http://www.r-project.org). The slideAnalyses function was used to generate windows varying in size from 100 to 230 base pairs (bp). Windows were shifted along the length of the COI alignment using 10 bp intervals. The top two mini-barcode fragments for each window length were selected for further analyses based on: (1) high mean Kimura 2-parameter (K2P) distance; (2) few zero pairwise non-conspecific distances; and (3) high proportion of clades shared between the neighbor-joining tree from the full-length DNA sequence alignment and the tree constructed using only data from selected windows. From this analysis, a total of 28 potential mini-barcode alignments were created.

Maximum likelihood analysis was conducted on the 29 datasets (1 full-length reference dataset and 28 SWAN mini-barcodes) using Garli 0.951 [34]. In all analyses, the K2P model of sequence evolution [35] was implemented as this is the model implemented on BOLD. The 28 mini-barcode maximum likelihood trees were then compared to the full-length reference tree using Ktreedist 1.0 [36]. The Ktreedist calculated K-scores (topology and branch length differences) and Robinson-Fouls symmetric difference (topological differences). For both methods, lower values indicated a high degree of similarity between the reference tree and the mini-barcode tree.

A DNA barcode gap analysis was conducted on the top-scoring mini-barcode dataset. Intra- and interspecific genetic distances were calculated using the K2P nucleotide substitution model in MEGA 6.0 and plotted. The maximum intraspecific distance was subtracted from the minimum interspecific distance to determine the barcoding gap [37] and the Jeffries-Matusita distance (J-M) statistic was used to test whether the intra- and interspecific genetic distance classes were separable. The J-M statistic takes into consideration the distance between the means of the intra- and interspecific genetic distances, and the distribution of values from the mean [38]. The J-M distance is asymptotic to 1.414 and as such, a value of 1.414 or greater suggests that intra- and interspecific genetic distances are statistically separable [39].

Primers were designed flanking the top-scoring mini-barcode region (LobsterMinibarF:5’-GGWGATGAYCAAATTTAYAAGT–3’ and LobsterMinibarR: 5’-CCWACTCCTCTTTCTACTATTCC –3’). Amplification and sequencing success were tested on both adult and phyllosoma samples of different lobster species. The adult samples included: two from the family Nephropidae (*Metanephrops mozambicus*, *Nephropsis stewarti*), two from Scyllaridae (*Scyllarides elisabethae*, *Scyllarides squammosus*), six from Palinuridae, comprising of three genera, namely; *Panulirus* (*P*. *homarus*, *P*. *versicolor*), *Palinurus* (*P*. *gilchristi*, *P*. *delagoae*) and *Jasus* (*J*. *lalandii*, *J*. *paulensis*) and one from the family Polychelidae (*Polycheles typhlops*). The phyllosoma samples included three specimens from the family Palinuridae (*Panulirus ornatus*, *P*. *homarus*, and *P*. *homarus rubellus*) and five from the family Scyllaridae (*Scyllarus arctus*, *Petrarctus rugosus*, *Acantharctus ornatus*, *Scyllarus sp*., and *Petractus sp*.). These samples provided 67% coverage across the different families within the lobster taxonomy.

DNA from 17 species (adults and larvae combined = 19 samples) was extracted from pereiopod tissue using the Zymo Quick-DNA Universal Kit (Zymo Research), as per the manufacturer’s protocol which was modified to include an initial incubation step at 55°C overnight. PCR reactions were 25 μl in volume and contained 30 ng genomic DNA, 12.5 μl OneTaq Quick-Load Master Mix (1X, BioLabs, New England), 0.50 μl forward and reverse mini-barcode primer (10 μM each), 6.5 μl sterile nuclease-free water, 2 μl additional MgCl_2_ (25 μM) and 2 μl Bovine Serum Albumin (BSA) (1 mg.m^-1^) was added. All PCR reactions were run with a negative control. The thermal cycling program included initial denaturation at 94°C for 2 minutes, followed by 35 cycles of denaturation at 94°C for 30 seconds, annealing at 46°C for 30 seconds, and extension at 68° C for 1 minute. The final extension step was carried out at 68°C for 5 minutes. PCR clean-up and sequencing reactions were performed at the Central Analytical Facilities (CAF) at the University of Stellenbosch (South Africa). All sequences were checked for their species specificity using the nucleotide BLAST tool (BLASTn) on NCBI GenBank. Percentage identification between 92–100% was used to confirm the exact species match.

The amplification success of the lobster-specific mini-barcode primer set was compared to that of the standard COI primer set (expected size = 658 bp) [40], a universal mini-barcode primer (expected size = 130 bp) [24], and internal COI mini-barcode primers (expected size = 313–319 bp) [21] (see S1 Table). A graphical representation with the relative annealing sites and the orientation of each primer set on the COI barcode region can be seen in S1 Fig. The internal mini-barcode primers designed by Leray *et al*. (2013) works in conjuncture with the Folmer *et al*. (1994) COI primers. PCR reactions were the same as above. Thermal cycling conditions can be found in S2 Table. The PCR products were visualized on a 1.2% (w/v) TBE agarose gel containing 0.02% Ethidium Bromide (EtBr). A 100 bp molecular weight marker (Solis Biodyne) was used to estimate the size of PCR products. PCR clean-up and sequencing were carried out on successful amplifications.

## Results

Smaller window sizes (100–160 bp from SWAN) had higher mean K2P distances and lower zero non-conspecific values in the K2P distance matrix. Larger window sizes (170–230 bp) showed better congruence of neighbor-joining trees (S3 Table). Larger mini-barcode fragments generated lower K- and R-F scores when compared with the reference tree (S4 Table). Based on these results, Fragment 230_b (position 109–339 of the full alignment) was selected as the best candidate for a mini-barcode.

**Fig 1:**
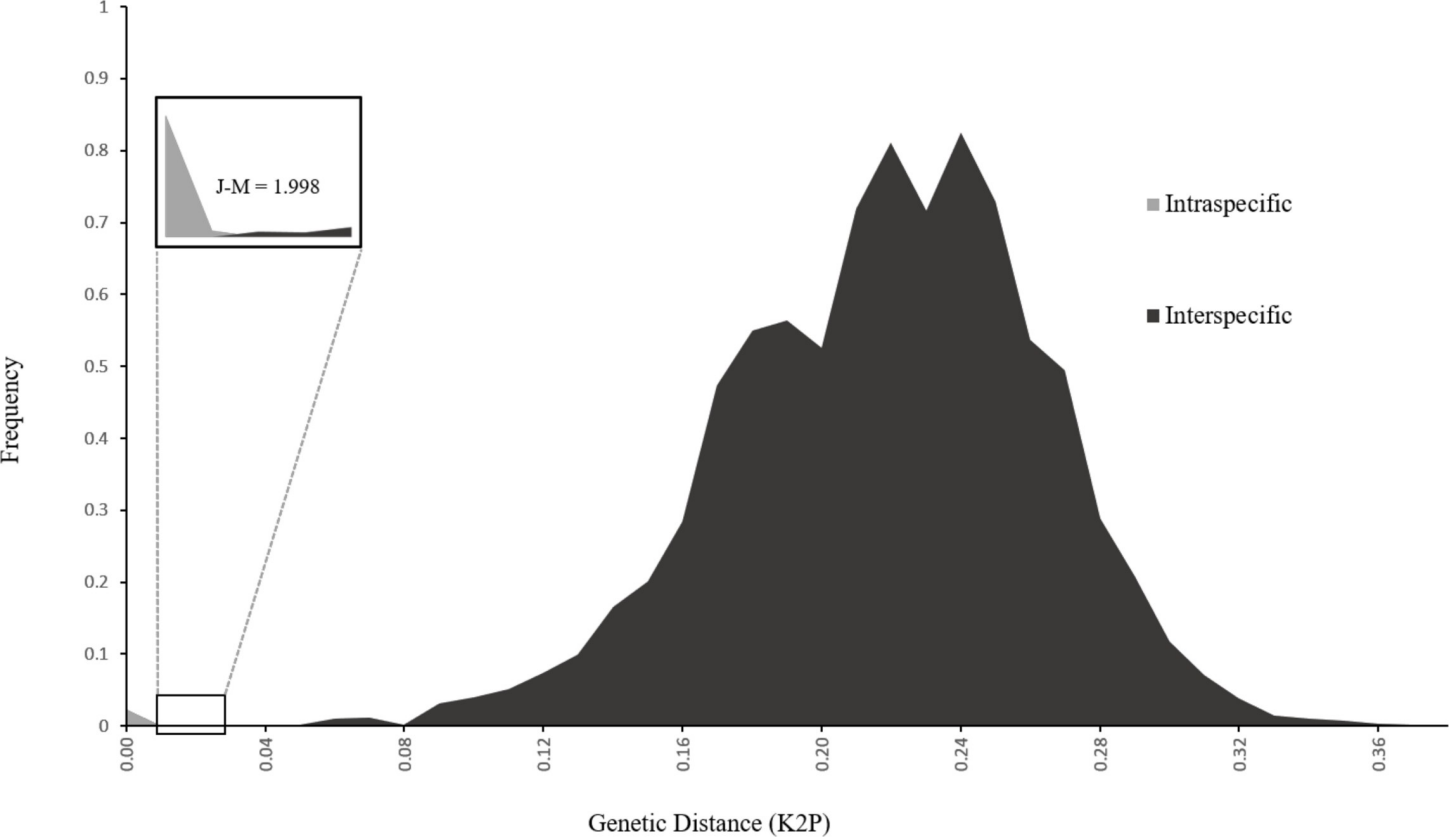
Frequency distributions of intra- and interspecific pairwise K2P distances calculated using the selected mini-barcode region (Fragment 230_b). The barcode gap (insert) lies between the genetic distances of 0.01 and 0.02.

The DNA barcode gap analysis was carried out on 350 DNA barcodes (S1 Spreadsheet) which included two representative individuals per species. To test the impact of sample size per species, the analysis was also carried out on a larger dataset with 2–5 individuals per species (S2 Fig). Increasing the sample size did not significantly impact the DNA barcode gap analysis. The intra-specific K2P pairwise distances in the Fragment 230_b alignment ranged from 0.00 to 0.01, while the inter-specific distances ranged from 0.02 to 0.36 (Fig 1). The position of the DNA barcode gap is between 0.01 and 0.02. The Jeffries-Matusita distance of 1.998 exceeds the significance thresholds and confirms that the intra- and interspecific distance classes are statistically separable.

The mini-barcode region was successfully amplified using the designed primers (LobsterMinibarF and LobsterMinibarR) across all 17 different species in both adult and phyllosoma samples (S3 Fig). BLAST search results confirmed that 16 mini-barcode sequences were a match (percentage identification between 92–100% was used to confirm the exact species match) to the morphologically identified adult and phyllosoma lobster voucher specimens. In three cases (*N*. *stewarti*, *Scyllarus sp*. and *Petrarctus sp*.) no direct match could be found, because no sequences were available on GenBank for these species. GenBank accession numbers are provided in S2 Spreadsheet.

**Fig 2:**
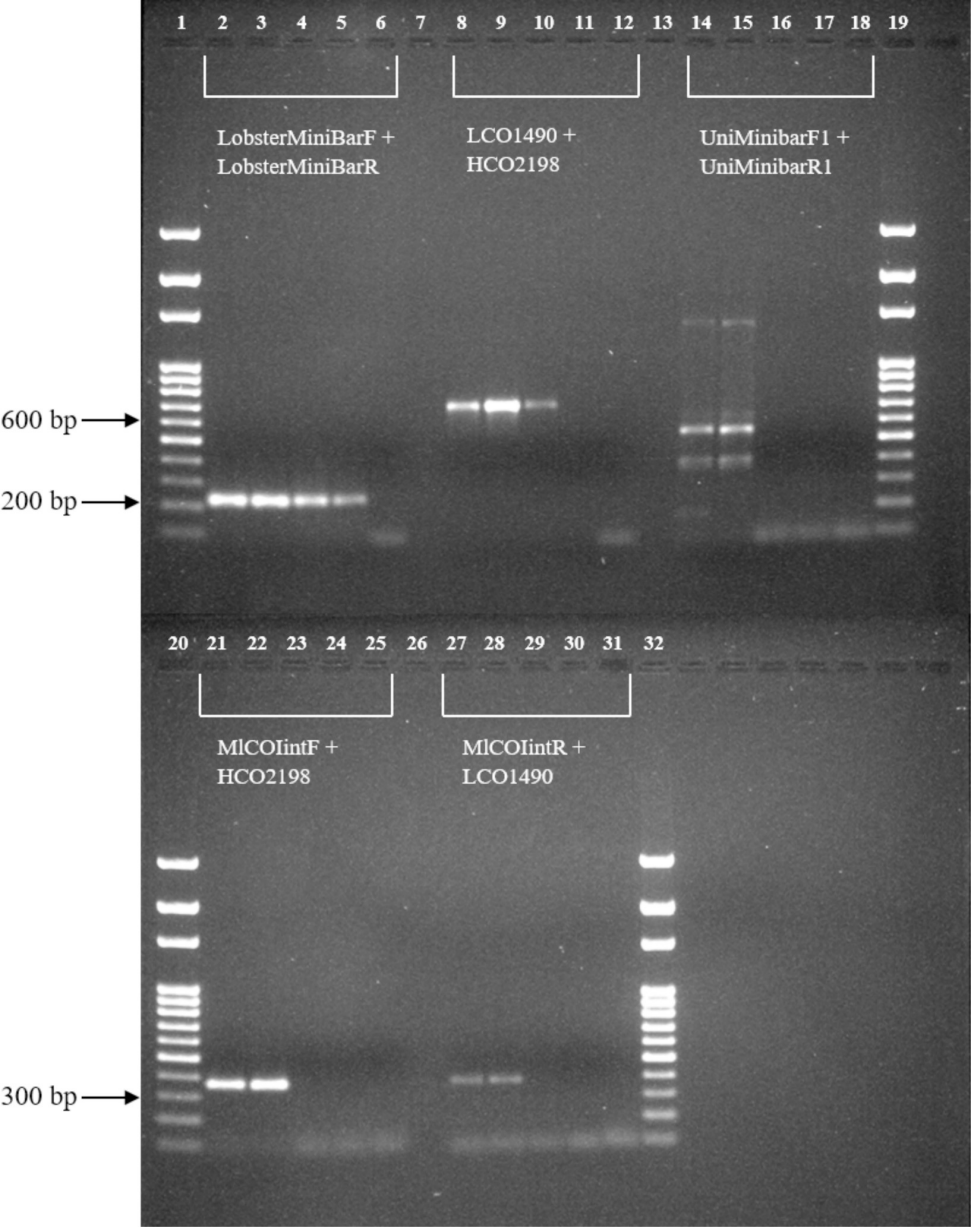
Amplification of four different cytochrome oxidase (COI) primer pairs tested in this study. Lanes 2, 3, 8, 9, 14, 15, 21, 22, 27 and 28 are PCR products recovered from DNA extracted from adult *Panulirus homarus*. Lane 4, 5, 10, 11, 16, 17, 23, 24, 30 and 31 are PCR products recovered from DNA extracted from phyllosoma samples. Lane 6, 12, 18, 25 and 31 are PCR negative controls. Lane 1, 19, 20 and 32 are 100 bp molecular weight marker (Solis Biodyne). Lane 7, 13 and 26 had no samples loaded.

PCR amplification was successful for all published primer pairs when tested on DNA extracted from adult *P*. *homarus* samples (Fig 2), but for phyllosomas, only the Folmer et al. (1994) primer set (LCOI490, HCO2198) produced a PCR product, for one of two specimens. In contrast, the lobster mini-barcode primers consistently amplified the DNA extracted from phyllosoma samples. BLAST searches performed on successfully amplified PCR products confirmed that all sequences were *P*. *homarus*, with a match of 92–99% (S5 Table). Given that our mini-barcode primer set was selected to amplify the most variable portion of the COI gene, the < 100% sequence match with data on GenBank is probably due to a combination of genetic diversity within species and limited COI data for these species currently uploaded. As more data from more species and populations are uploaded to GenBank or BOLD species identification will become more accurate.

## Discussion

The lobster specific mini-barcode primer pair designed in this study was tested on a taxonomically diverse set of marine lobsters from seven different genera. It consistently amplified COI from both adults and phyllosomas across all taxa and outperformed published primers. Confirmation of a barcode gap highlights its value as a diagnostic tool that can be used to match phyllosomas to species [37]. The sliding window analysis method [32] accurately identified the shortest, most informative portion of the COI gene of marine lobsters.

The taxon-specific mini-barcode primers were designed in response to the repeated low amplification success rate of the standard COI gene region in DNA extracted from lobster phyllosomas. Given our broader objective, to design primers that can account for the presence of marine lobsters using metabarcoding on unsorted zooplankton samples, a high amplification success for lobster larvae was considered to be crucial. The phyllosoma samples tested in this study were obtained from plankton tows at sea and stored in absolute ethanol. COI often failed to amplify completely using published primers, or the sequences obtained were messy and terminated abruptly. The effects of suboptimal field sampling conditions (temperature and pH fluctuations and contamination) [13], post-survey sorting of plankton samples, or inadequate preservation of biological material (whole phyllosomas) prior to DNA extraction may have contributed to the degradation or fragmentation of DNA [14, 41].

Other studies have had similar difficulties with the amplification of COI from larval material using different organisms. A study based on larval Antarctic marine invertebrates which were alcohol-preserved encountered a low amplification success of 22%, despite using 18S RNA, COI and 16S RNA primer sets [42]. The authors suggested using a taxon-specific primer to increase PCR amplification success rate. Baird *et al*. (2011) created a COI reference library for freshwater benthic macroinvertebrate specimens ranging in preservation time between <1 and 23 years old, but this yielded only 2.9% full-length usable barcodes [43]. Adding a universal mini-barcode primer increased the yield to 17.5%, and it was concluded that the DNA was likely degraded, because samples were collected and fixed in formalin in the field and thereafter transferred to 70% ethanol for long-term storage.

Hajibabaei *et al*. (2006) used both *in silico* and *in vitro* tests to examine the accuracy of mini-barcodes in species identification of century-old museum samples. Mini-barcodes of varying lengths were tested *in silico* on Australian fish and lepidoptera sequences and found to be as accurate as full-length barcodes. *In vitro* tests were subsequently carried out on museum specimens with varied age, preservation methods and taxonomic scope. Primers designed for the mini-barcodes had a success rate of > 90% after sequencing, compared to 50% in full-length primers. Hence mini-barcode primers which amplify a smaller region of COI can improve barcoding success where DNA is degraded.

The lobster mini-barcode designed in this study returned a higher amplification success for lobster phyllosomas than the universal mini-barcode [24]. Internal mini-barcodes (Leray *et al*. 2013) designed to work in conjunction with the commonly used COI primer set [40] were also tested in addition to degenerate versions of the universal COI primer set [44, 45]. The forward internal primer used in combination with the reverse COI primer and its degenerate versions had the highest amplification success. Nevertheless, when tested on lobster phyllosomas in the present study, these internal primers failed to amplify COI.

The emergence of metabarcoding techniques in combination with high throughput next-generation sequencing provides a powerful new tool for biodiversity assessments from environmental samples [46]. DNA metabarcoding can increase the speed, accuracy and resolution of species identification while allowing for cost-effective biodiversity monitoring. For example, zooplankton in the marine environment (including phyllosomas of various lobster species) are model organisms for monitoring trends in ecosystem health and biodiversity in the face of climate change and habitat degradation, because they exhibit rapid response to environmental change [47]. Within this context, taxa that are important to fisheries (i.e. decapods such as marine lobsters, crabs and prawns, or fish species important to fisheries) can be selected as indicator species when analysing mixed zooplankton samples.

The efficiency and accuracy of metabarcoding for taxonomic detection and identifications rely on specifically targeted barcodes which are taxonomically informative [48], and on suitable primer sets for amplifying hypervariable DNA regions from target organisms [21]. The method used to develop mini-barcodes for lobsters in our study can easily be applied to other taxa–for example crabs, shrimps or fish. Identifying the shortest, most variable portions of the genome are particularly relevant in applications involving next-generating sequencing technologies, such as Illumina, with limited read length.

To conclude, studies have highlighted the need for multiple metabarcoding assays to catalogue biodiversity, including universal and multiple taxon-specific assays [28]. From this perspective, the use of taxon-specific mini-barcodes is encouraged, because, in combination, they can maximize richness estimates and increase the possibility of recovering amplicons from degraded DNA [24, 28].

## Supporting information

S1 Spreadsheet Lobster COI sequences downloaded from GenBank and BOLD. (XLSX)

S1 Table Primer information table for the standard COI region, universal mini-barcode, internal COI mini-barcode and lobster mini-barcode. (PDF)

S1 Fig A graphical representation of the relative annealing sites and orientation of the different primer sets on the COI barcode region. (PDF)

S2 Table Thermal cycling conditions for the standard COI, universal mini-barcode (touch up PCR), internal COI mini-barcode (touch down PCR) primers and lobster mini-barcode. (PDF)

S3 Table Summary statistics of the sliding window analysis for two selected fragments of each fragment length, showing potential segments for mini-barcodes and their position within the full alignment. Statistics include mean Kimura 2-parameter (K2P) distance, proportion of zero non-conspecific K2P distance, proportion of zero cells in K2P distance matrix, and congruence of neighbour joining trees (clade composition and clade composition shallow).
(PDF)

S4 Table Summary statistics for comparison trees of all 28 fragments. K-scores and Robinson-Foulds (R-F) scores are used to identify best comparison trees. Each score is ranked based on the dataset in ascending order.
(PDF)

S2 Fig Frequency distributions of intra- and interspecific pairwise K2P distances calculated using the selected mini-barcode region (Fragment 230_b). The barcode gap (insert) lies between the genetic distances of 0.02 and 0.03.
(PDF)

S3 Fig Agarose gel image showing the amplification success of the new lobster mini-barcode primer to amplify a range of different adult and phyllosoma lobster samples. (PDF)

S2 Spreadsheet List of adult and phyllosoma samples amplified with the lobster mini-barcode primer set with detail data (taxonomy and GenBank accession numbers). (XLSX)

S5 Table Summary of amplification and sequencing results for each primer pair. PCR products that showed a single sharp band of correct size were sent for sequencing. Sequences were then BLASTed. Blast search results, identification % and E-value were recorded.
(PDF)
