# Supplementary material for: The design and testing of mini-barcode markers in marine lobsters
Source: PLoS One. 2019 Jan 24;14(1):e0210492. doi: 10.1371/journal.pone.0210492 (PMC6345471; doi:10.1371/journal.pone.0210492)
Supplement: S1 Table — (PDF) [file pone.0210492.s002.pdf]

S2 Table. Primer information table for the standard COI region, universal mini-barcode, internal COI mini-barcode and lobster mini-barcode.

Primers	Forward	Reverse	Expected size
**Standard COI**	LCO1490 5′- GGTCAACAAATCAT AAAGATATTGG-3′	HCO2198 5′- TAAACTTCAGGGTG ACCAAAAAATCA-3′	658 bp
**Universal mini-barcode**	UniMinibarF1 ' - TCCACTAATCACAARGATATTG GTAC-3'	UniMinibarR1 5'- GAAAATCATAATGAAGGCATGA GC-3'	130 bp
**Internal COI mini-barcode**	mlCOIintF 5′- GGWACWGGWTGAACWGTWTA YCCYCC-3′	mlCOIintR 5′- GGRGGRTASACSGTTCASCCSGT SCC-3′′	313 – 319 bp
**Lobster mini-barcode**	LobsterMinibarF 5′- GGWGATGAYCAAATTTAYAAGT -3′	LobsterMinibarR 5′- CCWACTCCTCTTTCTACTATTCC -3′	230 bp
