# Supplementary material for: The design and testing of mini-barcode markers in marine lobsters
Source: PLoS One. 2019 Jan 24;14(1):e0210492. doi: 10.1371/journal.pone.0210492 (PMC6345471; doi:10.1371/journal.pone.0210492)
Supplement: S1 Fig — (PDF) [file pone.0210492.s003.pdf]

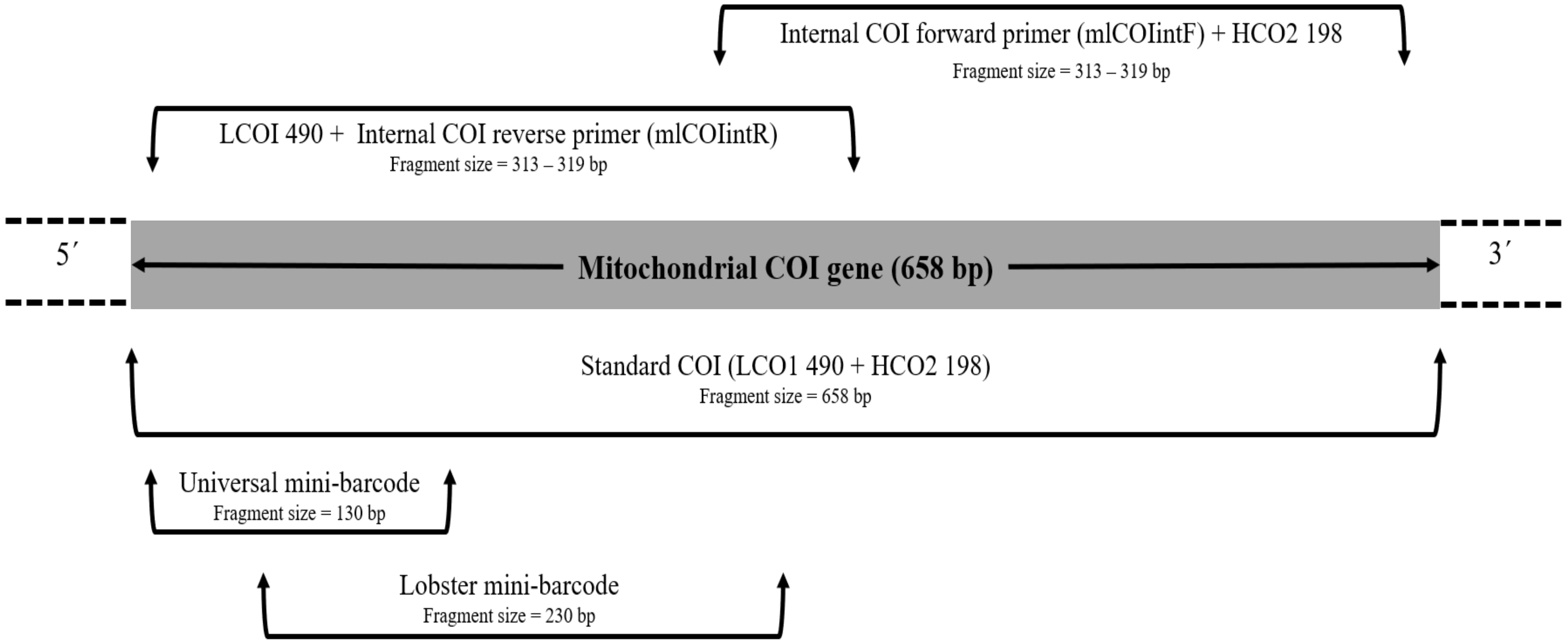

S3 Fig. A graphical representation of the relative annealing sites and orientation of the different primer sets on the COI barcode region.
