# Supplementary material for: The design and testing of mini-barcode markers in marine lobsters
Source: PLoS One. 2019 Jan 24;14(1):e0210492. doi: 10.1371/journal.pone.0210492 (PMC6345471; doi:10.1371/journal.pone.0210492)
Supplement: S2 Table — (PDF) [file pone.0210492.s004.pdf]

S4 Table: Thermal cycling conditions for the standard COI, universal mini-barcode (touch up PCR), internal COI mini-barcode (touch down PCR) primers and lobster mini-barcode.

	Standard COI			Universal mini-barcode (touch up)			Internal COI mini-barcode (touch down)			Lobster mini-barcode		
	**Cycles**	**° C**	**Min**	**Cycles**	**° C**	**Min**	**Cycles**	**° C**	**Min**	**Cycles**	**° C**	**Min**
**Initial denaturation**		94	2		94	2		94	2		94	2
**Denaturation**		94	1		94	1		94	10 s		94	30 s
**Annealing**	x 35	40	1	x 5	46	1	x 16	62 **	30 s	x 35	46	30 s
**Extension**		72	1.5		72	30 s		72	1		68	1
**Denaturation**		-	-		94	1		94	10 s		-	-
**Annealing**	-	-	-	x 35	53	1	x 25	46	30 s	-	-	-
**Extension**		-	-		72	30 s		72	1		-	-
**Final extension**		72	7		72	5		72	5		68	5

** Temperature (-1°C per cycle = touch down PCR)
