# Supplementary material for: The design and testing of mini-barcode markers in marine lobsters
Source: PLoS One. 2019 Jan 24;14(1):e0210492. doi: 10.1371/journal.pone.0210492 (PMC6345471; doi:10.1371/journal.pone.0210492)
Supplement: S3 Table — Statistics include mean Kimura 2-parameter (K2P) distance, proportion of zero non-conspecific K2P distance, proportion of zero cells in K2P distance matrix, and congruence of neighbour joining trees (clade composition and clade composition shallow). (PDF) [file pone.0210492.s005.pdf]

S5 Table. Summary statistics of the sliding window analysis for two selected fragments of each fragment length, showing potential segments for mini-barcodes and their position within the full alignment. Statistics include mean Kimura 2-parameter (K2P) distance, proportion of zero non-conspecific K2P distance, proportion of zero cells in K2P distance matrix, and congruence of neighbour joining trees (clade composition and clade composition shallow).

Fragment length/name	Position	K2P dist	Zero non con dist	Zero dist	Clade comp	Clade comp shallow
Fragment100_a	87	0.295	0.0714	0.00298	0.652	0.966
Fragment100_b	152	0.282	0.0800	0.00300	0.618	0.954
Fragment110_a	84	0.277	0.0714	0.00296	0.644	0.966
Fragment110_b	149	0.278	0.0686	0.00291	0.647	0.966
Fragment120_a	84	0.278	0.0714	0.00295	0.658	0.966
Fragment120_b	154	0.281	0.0600	0.00288	0.681	0.966
Fragment130_a	85	0.278	0.0714	0.00296	0.647	0.966
Fragment130_b	150	0.274	0.0600	0.00287	0.670	0.966
Fragment140_a	84	0.270	0.0714	0.00291	0.655	0.966
Fragment140_b	155	0.270	0.0600	0.00288	0.667	0.966
Fragment150_a	84	0.271	0.0714	0.00291	0.695	0966
Fragment150_b	156	0.267	0.600	0.00290	0.667	0.966
Fragment160_a	84	0.270	0.0714	0.00290	0.687	0.977
Fragment160_b	132	0.268	0.0571	0.00280	0.684	0.971
Fragment170_a	83	0.264	0.0714	0.00287	0.695	0.971

Fragment170_b	131	0.263	0.0571	0.00278	0.690	0.971
Fragment180_a	81	0.264	0.0714	0.00287	0.698	0.971
Fragment180_b	126	0.264	0.0571	0.00277	0.693	0.971
Fragment190_a	82	0.262	0.0600	0.00278	0.698	0.971
Fragment190_b	115	0.264	0.0543	0.00270	0.684	0.971
Fragment200_a	85	0.262	0.0486	0.00273	0.716	0.977
Fragment200_b	113	0.264	0.0429	0.00264	0.707	0.977
Fragment210_a	88	0.270	0.0486	0.00273	0.718	0.977
Fragment210_b	111	0.268	0.0429	0.00264	0.716	0.977
Fragment220_a	87	0.269	0.0486	0.00272	0.716	0.978
Fragment220_b	110	0.269	0.0429	0.00262	0.713	0.977
Fragment230_a	85	0.265	0.0486	0.00272	0.710	0.971
Fragment230_b	109	0.266	0.0429	0.00260	0.0716	0.977
