# Supplementary material for: The design and testing of mini-barcode markers in marine lobsters
Source: PLoS One. 2019 Jan 24;14(1):e0210492. doi: 10.1371/journal.pone.0210492 (PMC6345471; doi:10.1371/journal.pone.0210492)
Supplement: S4 Table — K-scores and Robinson-Foulds (R-F) scores are used to identify best comparison trees. Each score is ranked based on the dataset in ascending order. (PDF) [file pone.0210492.s006.pdf]

S6 Table. Summary statistics for comparison trees of all 28 fragments. K-scores and Robinson-Foulds (R-F) scores are used to identify best comparison trees. Each score is ranked based on the dataset in ascending order.

Comparison tree	Position	K-score	Scale factor	R-F score	K-score rank	R-F score rank
Fragment100_a	87	2.01237	0.76807	376	25	25
Fragment100_b	152	2.11378	0.12715	362	28	24
Fragment110_a	84	1.83492	1.37474	340	15	20
Fragment110_b	149	2.04018	0.12333	356	27	22
Fragment120_a	84	1.93939	0.55600	360	22	23
Fragment120_b	154	1.90771	0.26661	336	20	19
Fragment130_a	85	1.90815	0.21935	336	21	19
Fragment130_b	150	1.95615	0.16101	344	23	21
Fragment140_a	84	1.98095	0.27977	328	24	16
Fragment140_b	155	1.84495	0.58261	318	17	15
Fragment150_a	84	1.88476	0.16584	334	19	18
Fragment150_b	156	1.67155	0.54366	308	9	12
Fragment160_a	84	2.02150	0.13397	316	26	14
Fragment160_b	132	1.72608	0.57789	296	14	9
Fragment170_a	83	1.85863	0.18692	330	18	17
Fragment170_b	131	1.71813	0.25365	312	13	13
Fragment180_a	81	1.67349	0.56663	304	10	10
Fragment180_b	126	1.56501	0.88654	276	5	5

Fragment190_a	82	1.84444	0.23458	306	16	11
Fragment190_b	115	1.59154	1.00567	264	8	2
Fragment200_a	85	1.69298	0.61303	284	11	8
Fragment200_b	113	1.70125	0.88842	270	12	4
Fragment210_a	88	1.57009	0.77518	270	6	4
Fragment210_b	111	1.56155	0.82888	282	4	7
Fragment220_a	87	1.51802	0.75945	278	3	6
Fragment220_b	110	1.51785	0.80795	278	2	6
Fragment230_a	85	1.58463	0.76811	266	7	3
**Fragment230_b**	**109**	**1.47431**	**0.84953**	**258**	**1**	**1**
