# Supplementary material for: The design and testing of mini-barcode markers in marine lobsters
Source: PLoS One. 2019 Jan 24;14(1):e0210492. doi: 10.1371/journal.pone.0210492 (PMC6345471; doi:10.1371/journal.pone.0210492)
Supplement: S2 Fig — The barcode gap (insert) lies between the genetic distances of 0.02 and 0.03. (PDF) [file pone.0210492.s007.pdf]

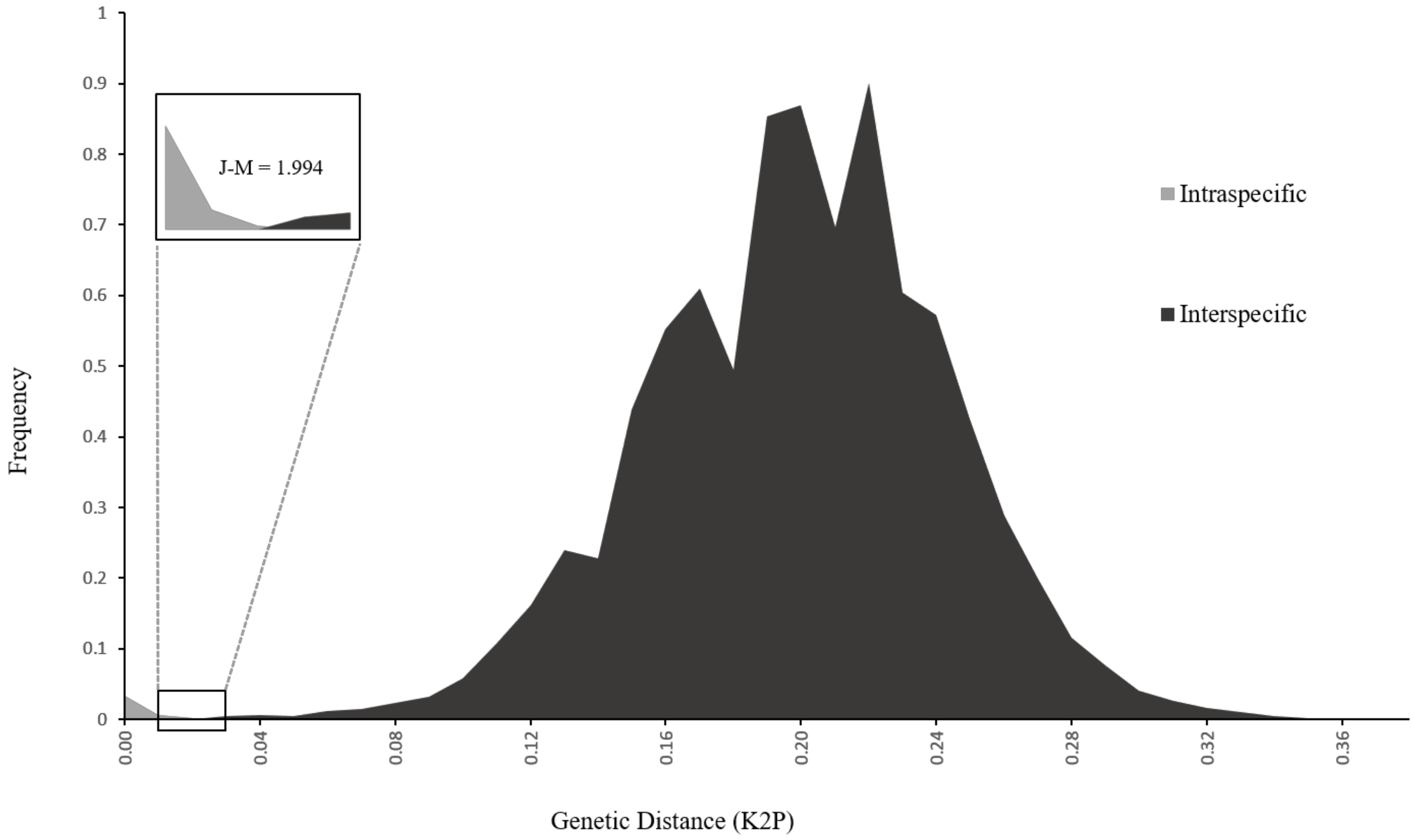

S7 Fig. Frequency distribution of intra- and interspecific pairwise K2P distances calculated using the selected mini-barcode region (Fragment 230_b). The barcode gap (insert) lies between the genetic distances of 0.02 and 0.03.
