# Supplementary material for: The design and testing of mini-barcode markers in marine lobsters
Source: PLoS One. 2019 Jan 24;14(1):e0210492. doi: 10.1371/journal.pone.0210492 (PMC6345471; doi:10.1371/journal.pone.0210492)
Supplement: S3 Fig — (PDF) [file pone.0210492.s008.pdf]

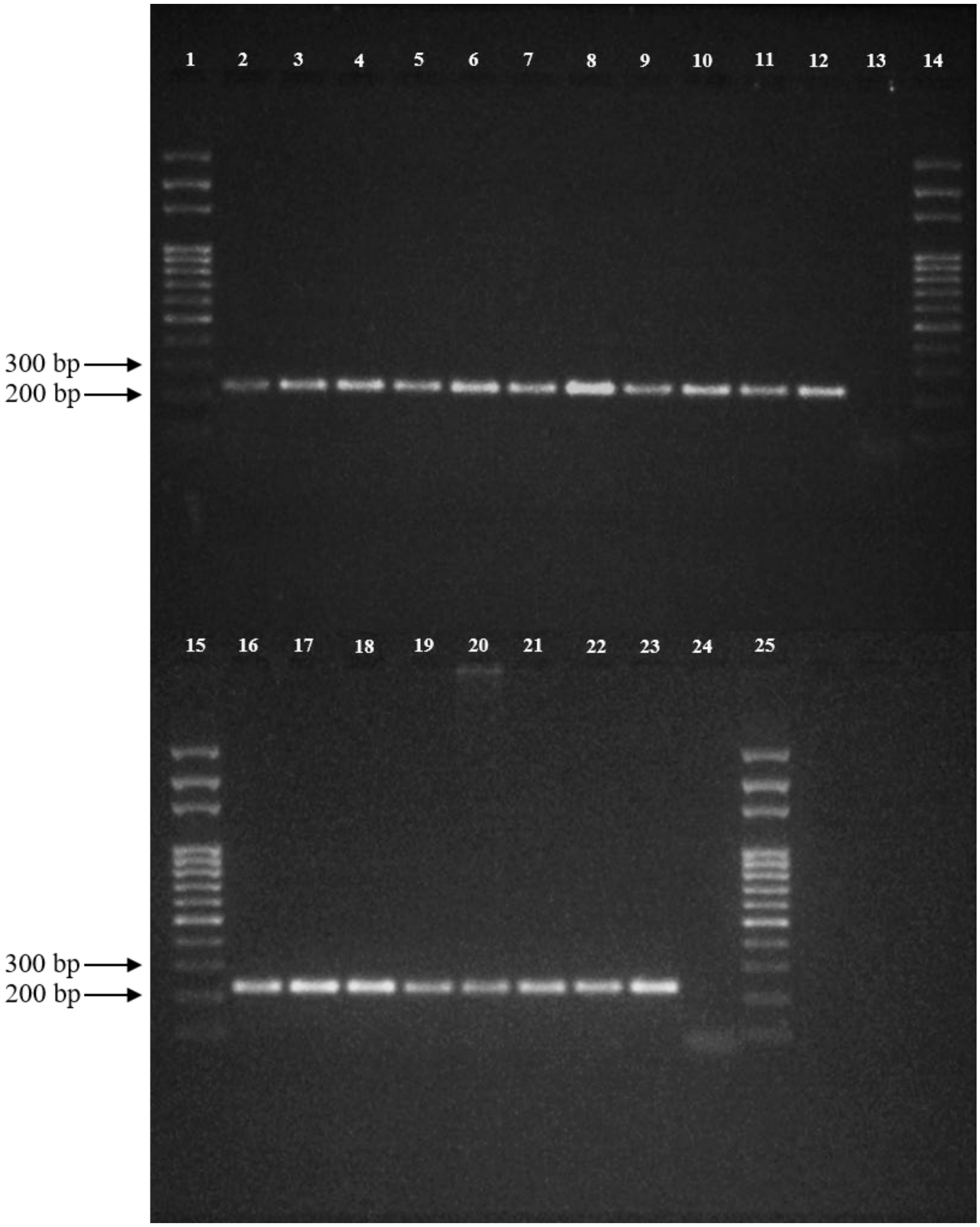

S8 Fig. Agarose gel image showing the amplification success of the new lobster mini-barcode primer to amplify a range of different adult and phyllosoma lobster samples. Amplification success rate of the newly designed lobster mini-barcode primer set across different adult and phyllosoma lobster samples. Lanes 2 to 12* are the PCR products recovered from the different adult lobster species. Lanes 16 to 23** are the PCR products recovered from the different

phyllosoma lobster species. Lane 13 and 24 are PCR negative controls. Lane 1, 14, 15 and 25 are 100 bp molecular weight marker (Solis Biodyne).

*Adult samples: Lane 2 - *Metanephrops mozambicus*, lane 3 - *Nephropsis stewarti*, lane 4 - *Scyllarides elisabethae*, lane 5 - *Scyllarides squammosus*, lane 6 - *Panulirus homarus*, lane 7 - *Panulirus versicolor*, lane 8 - *Palinurus gilchristi*, lane 9 - *Palinurus delagoae*, lane 10 - *Jasus lalandii*, lane 11 - *Jasus paulensis*, and lane 12 - *Polycheles typhlops*.

**Phyllosoma samples: Lane 16 - *Panulirus ornatus*, lane 17 - *Panulirus homarus*, lane 18 - *Panulirus homarus rubellus*, lane 19 - *Scyllarus arctus*, lane 20 - *Petrarctus rugosus*, lane 21 - *Acantharctus ornatus*, lane 22 - *Scyllarus sp*, and lane 23 - *Petractus sp.*.
