# Supplementary material for: The design and testing of mini-barcode markers in marine lobsters
Source: PLoS One. 2019 Jan 24;14(1):e0210492. doi: 10.1371/journal.pone.0210492 (PMC6345471; doi:10.1371/journal.pone.0210492)
Supplement: S5 Table — PCR products that showed a single sharp band of correct size were sent for sequencing. Sequences were then BLASTed. Blast search results, identification % and E-value were recorded. (PDF) [file pone.0210492.s010.pdf]

S10 Table. Summary of amplification and sequencing results for each primer pair. PCR products that showed a single sharp band of correct size were sent for sequencing. Sequences were then BLASTed. Blast search results, identification % and E-value were recorded.

Primer Pair	Sample	Blast top hit	Identification %	E-value
**LobsterMiniBarF + LobsterMiniBarR**	Adult 1	*Panulirus homarus*	97%	7e-80
	Adult 2	*Panulirus homarus*	96%	4e-82
	Phyllosoma 1	*Panulirus homarus*	93%	3e-74
	Phyllosoma 2	*Panulirus homarus*	92%	5e-71
**LCO1490 + HCO2198**	Adult 1	*Panulirus homarus*	96%	0.0
	Adult 2	*Panulirus homarus*	97%	0.0
	Phyllosoma 1	*Panulirus homarus*	97%	0.0
	Phyllosoma 2	No amplification	No amplification	No amplification
**UniMinibarF1 + UniMinibarR1**	Adult 1	Multiple bands	No amplification	No amplification
	Adult 2	Multiple bands	No amplification	No amplification
	Phyllosoma 1	No amplification	No amplification	No amplification

	Phyllosoma 2	No amplification	No amplification	No amplification
**MlCOIintF + HCO2198**	Adult 1	Multiple bands	No amplification	No amplification
	Adult 2	*Panulirus homarus*	99%	5e-148
	Phyllosoma 1	No amplification	No amplification	No amplification
	Phyllosoma 2	No amplification	No amplification	No amplification
**MlCOIintR + LCO1490**	Adult 1	Multiple bands	No amplification	No amplification
	Adult 2	*Panulirus homarus*	99%	5e-148
	Phyllosoma 1	No amplification	No amplification	No amplification
	Phyllosoma 2	No amplification	No amplification	No amplification
